# Responses to membrane potential-modulating ionic solutions measured by magnetic resonance imaging of cultured cells and in vivo rat cortex

**DOI:** 10.7554/eLife.101642

**Published:** 2025-07-03

**Authors:** Kyeongseon Min, Sungkwon Chung, Seung-Kyun Lee, Jongho Lee, Phan Tan Toi, Daehong Kim, Jung Seung Lee, Jang-Yeon Park

**Affiliations:** 1 Department of Electrical and Computer Engineering, Seoul National University Seoul Republic of Korea; 2 Department of Physiology, Sungkyunkwan University School of Medicine Suwon Republic of Korea; 3 Department of Biomedical Engineering, Sungkyunkwan University Seoul Republic of Korea; 4 Department of Intelligent Precision Healthcare Convergence, Sungkyunkwan University Seoul Republic of Korea; 5 National Cancer Center Goyang-si Republic of Korea; University of Oxford Oxford United Kingdom; University of Cambridge Cambridge United Kingdom

**Keywords:** magnetic resonance imaging, membrane potential, T2 relaxation time, magnetization transfer, SH-SY5Y, jurkat, Rat, Other

## Abstract

Membrane potential plays a crucial role in various cellular functions. However, existing techniques for measuring membrane potential are often invasive or have limited recording depth. In contrast, MRI offers noninvasive imaging with desirable spatial resolution over large areas. This study investigates the feasibility of utilizing MRI to detect responses of cultured cells and in vivo rat cortex to membrane potential-modulating ionic solutions by measuring magnetic resonance parameters. Our findings reveal that depolarizing (or hyperpolarizing) ionic solutions increase (or decrease) the *T*_2_ relaxation time, while the ratio of bound to free water protons shows the opposite trend. These findings also suggest a potential approach to noninvasively detect changes in membrane potential using MRI.

## Introduction

Membrane potential is a fundamental property of all living cells, influencing crucial cell functions (Abdul Kadir et al., 2018) such as neuronal and myocyte excitability, volume control, cell proliferation, and secretion. In the fields of neuroscience, membrane potential is of significant importance, as neural activities arise from the dynamic propagation of this electric potential. From a clinical perspective, deviations from normal membrane potential levels contribute to various diseases, including seizures (Jefferys, 1995), arrhythmia (Helfant, 1986), and hypoglycemia (Kane et al., 1996). Given its scientific and clinical importance, effective methods to detect changes in membrane potential have long been sought.

The intracellular recording technique using sharp glass electrodes is a commonly used method to detect changes in membrane potential (Ling and Gerard, 1949; Neher and Sakmann, 1976). It provides real-time absolute recordings of membrane potential. Optical imaging is another major approach to detect changes in membrane potential. Voltage-sensitive dyes enable voltage imaging at the cellular level (Tasaki et al., 1968). Fluorescent calcium indicators detect calcium transients associated with neuronal activation (Tsien, 1980). Label-free optical imaging techniques (Zhou et al., 2021) utilize various contrast mechanisms such as cell membrane deformation, which are directly coupled with changes in membrane potential. Despite their efficacy, these methods have limitations when applied to intact biological systems due to their invasive nature, requiring procedures such as craniotomy.

As noninvasive techniques for directly or indirectly detecting brain activation in vivo, several imaging modalities have been developed, including EEG (Berger, 1929), MEG (Cohen, 1968), and MRI (Mansfield, 1977). EEG and MEG detect electric potentials on the scalp and extracranial magnetic fields, respectively, which are directly induced by neuronal activity in the brain. Although these techniques are noninvasive and provide excellent temporal resolution of milliseconds or less, they are constrained by shallow imaging depth and spatial localization challenges (He et al., 2018).

In contrast, MRI enables noninvasive imaging with good spatial resolution of millimeters over a large brain volume, making it an appropriate tool for in vivo functional brain imaging. To date, the mainstream of functional MRI (fMRI) utilizes hemodynamic responses driven by brain activation, such as the blood oxygen level-dependent (BOLD) effect (Ogawa et al., 1990). However, while the BOLD contrast mechanism reflects dynamic changes in neuronal activity through neurovascular coupling, it provides inherently indirect and relatively slow, hemodynamic-responsive information of brain function (Logothetis et al., 2001). On the other hand, many studies have attempted to explore the possibility of using MRI to directly detect neuronal activity (Bandettini et al., 2005; Roth, 2023). These studies utilized neuronal current-dependent signal phase shifts (Petridou et al., 2006; Wijesinghe and Roth, 2009; Sundaram et al., 2016) and magnitude decay (Kamei et al., 1999; Xiong et al., 2003; Chow et al., 2006; Truong et al., 2019), the Lorentz effect (Truong and Song, 2006), high temporal resolution (Sundaram et al., 2010; Toi et al., 2022), ghost artifacts (Paley et al., 2009), or cell swelling (Le Bihan et al., 2006; Bai et al., 2016), while concerns about sensitivity exist (Chu et al., 2004; Konn et al., 2004; Parkes et al., 2007; Miller et al., 2007; Roth and Basser, 2009; Luo et al., 2009).

In this study, we investigated the possibility of using MRI to detect membrane potential changes induced by modulating ionic solutions. Specifically, we explored how *T*_2_ relaxation time and magnetization transfer (MT) correlate with membrane potential changes, both in vitro and in vivo. In vitro experiments utilized two homogeneous and electrically non-active cells, that is neuroblastoma (SH-SY5Y) and leukemia (Jurkat) cell lines, providing a controlled environment free from hemodynamic effects. This aided in accurately evaluating changes in MR parameters with membrane potential. In vivo experiments, conducted on a rat model with a craniotomy-exposed cortex, aimed to reproduce the in vitro findings.

## Results

### In vitro changes in MR parameters induced by membrane potential

**Figure 1.**
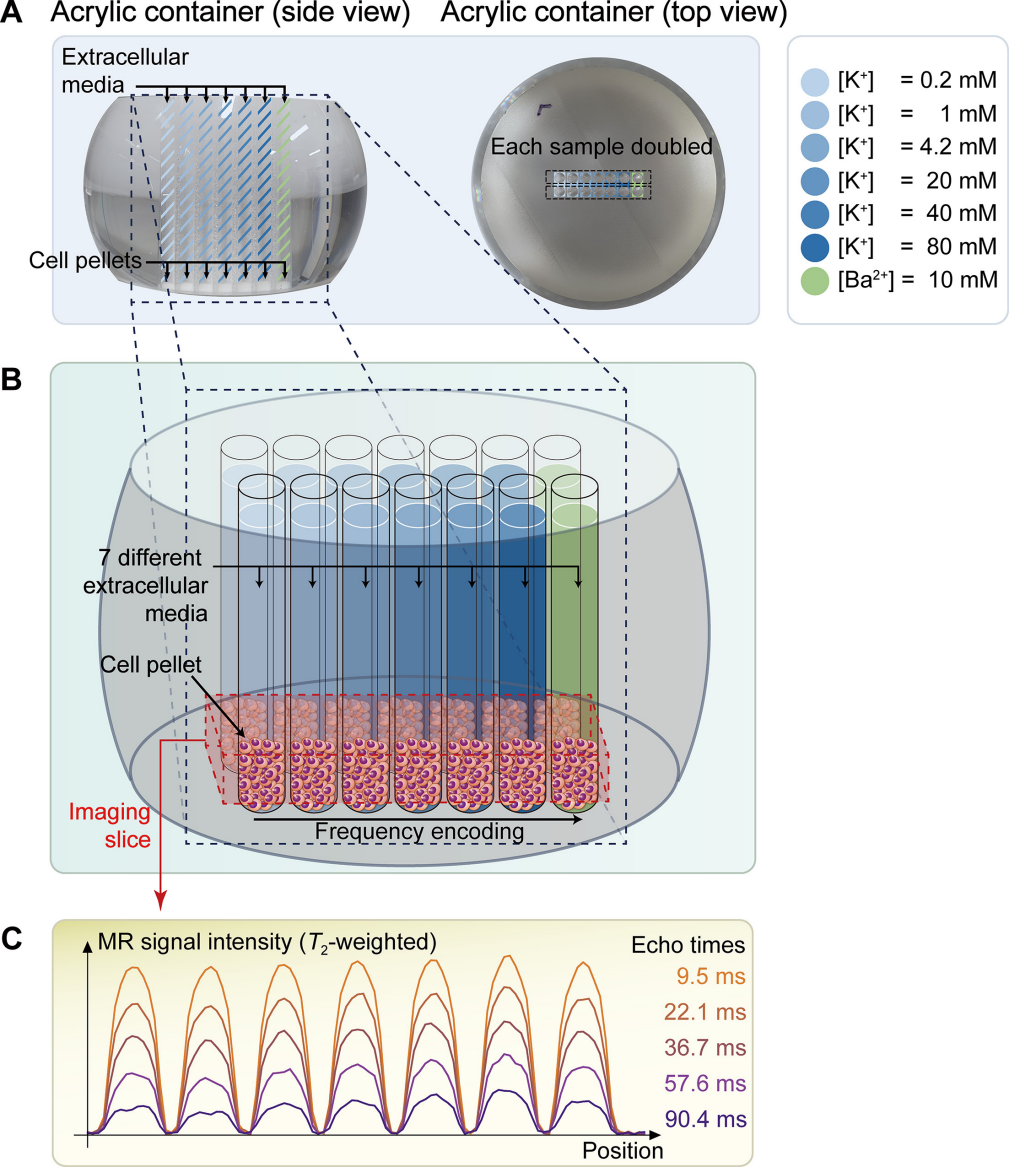
The schematic diagram of the in vitro experiment. (**A**) The picture on the left displays a side view of a double-sided cut spherical acrylic container with fabricated wells filled with extracellular media and cell pellets. As depicted in the top-view picture on the right, fourteen wells (matrix = 2 × 7) were created on the acrylic container, allowing each of the seven samples with six different K^+^ concentrations ([K^+^]=0.2–80 mM) and one Ba^2+^ concentration ([Ba^2+^]=10 mM) to be doubled in the same column for improved signal-to-noise ratio (SNR) in MR signal acquisition. (**B**) The image illustrates the configuration after loading cells into the wells and pelleting them at the bottom of the wells. The imaging slice was positioned 0.5 mm below the pellet-media interface to acquire signals predominantly from the cell pellets. (**C**) Representative one-dimensional *T*_2_-weighted MR signals with 5 selected echo times out of a total of 50 acquired echo times.

A non-excitable neuroblastoma cell line, SH-SY5Y, was selected to investigate the relationship between MR parameters and membrane potential modulated by ionic solutions. After culturing SH-SY5Y cells, they were suspended in extracellular media with various potassium ion concentrations ([K^+^]), while maintaining constant osmolarity by adjusting [Na^+^]. As the control condition, [K^+^]=4.2 mM was selected. For depolarization conditions, [K^+^]=20, 40, and 80 mM were used. For hyperpolarization conditions, [K^+^]=0.2 and 1 mM were used. The suspended cells were concentrated with centrifugation in an acrylic container and scanned in a 9.4T preclinical MRI system. The imaging slice was positioned 0.5 mm below the pellet-extracellular media interface to ensure that signals were acquired from the cell pellet. Under each condition, *T*_2_ and MT parameters such as pool size ratio (PSR) and magnetization transfer rate (*k_mf_*) were measured (Figure 1). The PSR value represents the ratio of hydrogen protons in macromolecules and free water, and *k_mf_* represents the magnetization transfer rate of hydrogen protons from macromolecules to free water. In addition, the membrane potential of SH-SY5Y cells in each condition was separately measured via patch clamp recording.

**Figure 2.**
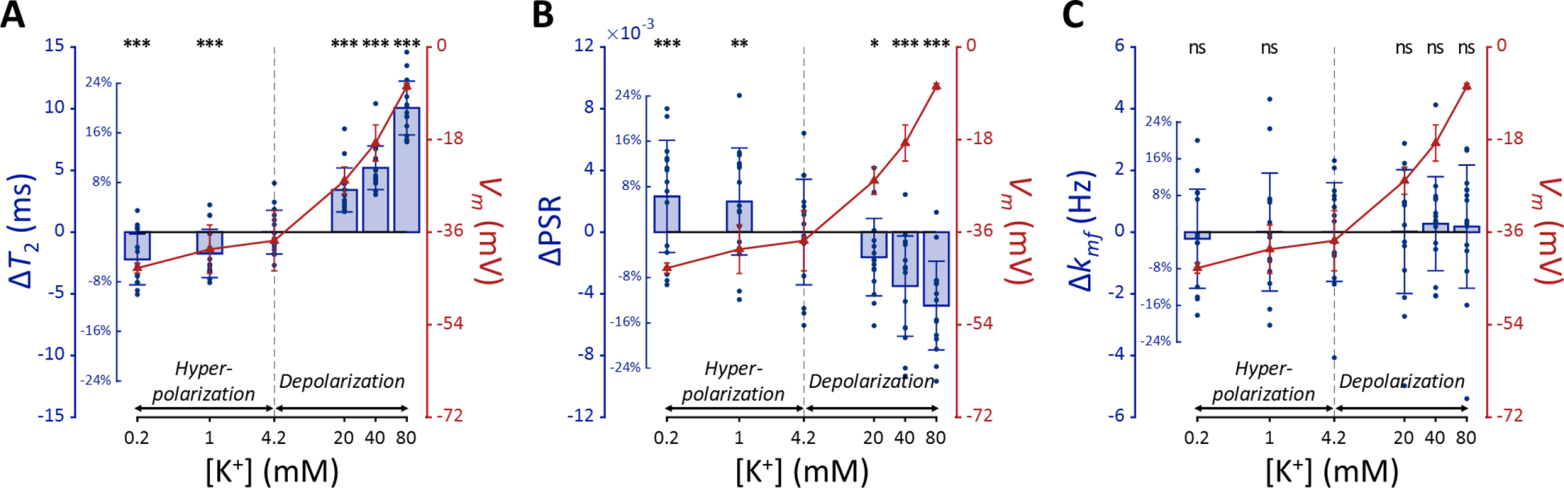
MR parameters and membrane potential (*V_m_*) of SH-SY5Y cells versus extracellular K^+^ concentrations ([K^+^]). Changes in (**A**) *T*_2_, (**B**) PSR, and (**C**) *k_mf_* are displayed with blue bars (n=15). Membrane potentials are plotted with red triangles (n=3). The abscissa is in logarithmic scale. Error bars denote standard deviation. Statistical significance of changes in MR parameters is marked with asterisks (ns: p>0.05, *: p<0.05, **: p<0.01, ***: p<0.001).

The changes of *T*_2_, PSR, and *k_mf_* in SH-SY5Y cells when the membrane potential (*V_m_*) was modulated by varying [K^+^] are shown in Figure 2, alongside the actual *V_m_* measured via patch clamp recordings. We conducted statistical analyses to assess the effect of changes in *V_m_* from the control condition (Δ*V_m_*) on the MR parameters. A linear mixed-effect model was applied to account for inter-sample variability and repeated measurements. This model included MR parameters as dependent variables, Δ*V_m_* as a fixed effect, and cell batch as a random effect. The analysis yielded the following relationships:

(1) $$\displaystyle  T_2\ (\text{ms}) = 49.7 \left(1 + 0.00687\, \Delta V_m\right)$$

(2) $$\displaystyle  \text{PSR} = 0.0377 \left(1 - 0.00542 \, \Delta V_m \right)$$

(3) $$\displaystyle  k_{\text{mf}}\ (\text{Hz}) = 14.8 \left(1 + 0.000648\, \Delta V_m\right)$$

The effects of Δ*V_m_* on *T*_2_ and PSR were both significant (p<0.0001), indicating an increase in *T*_2_ and a decrease in PSR during depolarization at high [K^+^], with the opposite trend during hyperpolarization at low [K^+^]. On the other hand, the effect of Δ*V_m_* on *k_mf_* was not significant (*P*=0.360).

Subsequent post-hoc analyses compared each experimental condition to the control using Dunnett’s test to account for multiple comparisons (Figure 2). During depolarization induced by the highest [K^+^] (80 mM, Δ*V_m_* = 30.0 mV), changes in MR parameters were observed as a 20.0% increase in *T*_2_ (Δ*T*_2_=10.1ms, p<0.0001) and a 12.9% decrease in PSR (ΔPSR = −0.00476, p<0.0001). Conversely, during hyperpolarization induced by the lowest [K^+^] (0.2 mM, Δ*V_m_* = −5.33 mV), *T*_2_ decreased by 4.40% (Δ*T*_2_ = −2.21ms, p<0.0001) and PSR increased by 6.28% (ΔPSR = 0.00231, p<0.0005). However, changes in *k_mf_* were not significant across all conditions (p>0.05). These findings from in vitro SH-SY5Y cell experiments suggest that MR parameters, such as *T*_2_ and PSR, exhibit sufficient sensitivity to detect alterations in membrane potential induced by varying [K^+^], including both depolarization and hyperpolarization.

#### Using a K^+^ channel blocker

In this experiment, we investigated whether depolarization induced by altering potassium permeability with barium ions (Ba^2+^) would affect MR parameters similarly to depolarization induced by varying [K^+^], thereby further validating our findings. For this purpose, we administered barium ions (Ba^2+^) at a concentration of 10 mM to induce depolarization while maintaining constant osmolarity by adjusting [Na^+^]. Ba^2+^ was chosen because it inhibits several types of two-pore-domain potassium channels (Lesage and Lazdunski, 2000; Ma et al., 2011), which predominantly regulate the resting membrane potential. Previous studies have confirmed that Ba^2+^ depolarizes the membrane potential in SH-SY5Y and Jurkat cells (Vaughan et al., 1995; Pottosin et al., 2008).

**Figure 3.**
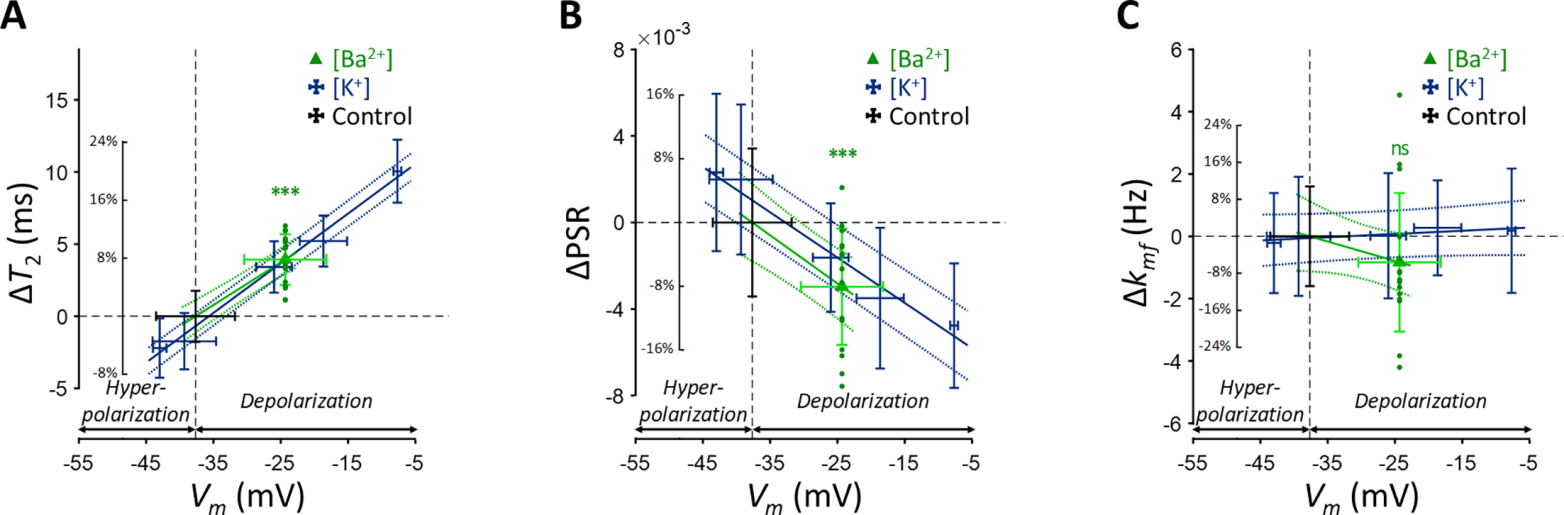
Changes in (**A**) *T*_2_, (**B**) PSR, and (**C**) *k_mf_* of SH-SY5Y cells across experimental conditions: [K^+^]=0.2–80 mM (blue cross) and [Ba^2+^]=10 mM (green triangle), compared to the control condition (black cross). Data from fifteen experiments (n=15) are displayed. Linear regression lines for [K^+^] data (blue solid line) and [Ba^2+^] data (green solid line) are drawn along with dotted lines representing 95% confidence intervals. Error bars denote standard deviation. Statistical significance of changes in MR parameters with [Ba^2+^]=10 mM is marked with asterisks (ns: p>0.05, *: p<0.05, **: p<0.01, ***: p<0.001).

The Ba^2+^-induced depolarization condition was compared with K^+^-induced depolarization and hyperpolarization conditions (Figure 3). To compare the effects of [Ba^2+^] and [K^+^] on MR parameters, a linear mixed-effect model was utilized. This model included MR parameters as dependent variables, Δ*V_m_* and its interaction with a group variable (indicating whether Δ*V_m_* was induced by [Ba^2+^] or [K^+^]) as fixed effects, and cell batch as a random effect. This analysis revealed no significant interactions for all MR parameters assessed (p=0.182 for *T*_2_, p=0.788 for PSR, and p=0.0890 for *k_mf_*). These findings suggest that changes in *T*_2_ and PSR by membrane potential do not depend on the specific method of altering the membrane potential, whether by varying [K^+^] or applying [Ba^2+^]. This implies that if the changes in MR parameters observed with varying [K^+^] were not primarily due to membrane potential but were due to a unique K^+^-related mechanism, then experiments using [Ba^2+^] would not result in similar changes.

Subsequent post-hoc analyses compared the [Ba^2+^]-induced depolarization to the control using Dunnett’s test to account for multiple comparisons (Figure 3). In response to depolarization caused by [Ba^2+^]=10 mM, *T*_2_ increased by 7.82% (Δ*T*_2_=3.93ms, p<0.0001) and PSR decreased by 8.06% (ΔPSR = −0.00297, p<0.0001). The change in *k_mf_* was not significant (Δ*k_mf_* = −0.832 Hz, p=0.263). The depolarization of membrane potential induced by [Ba^2+^] was measured as Δ*V_m_* = 13.3 mV by patch clamp recording.

#### Using another cell type

To investigate whether membrane potential-modulating ionic solutions produce similar changes in MR parameters across different cell types, we assessed another cell line, Jurkat, under the same experimental conditions applied to SH-SY5Y cells. These conditions included a control condition ([K^+^]=4.2 mM), hyperpolarization under decreased [K^+^] conditions ([K^+^]=0.2 and 1 mM), depolarization under increased [K^+^] conditions ([K^+^]=20, 40, and 80 mM), and a Ba^2+^-induced depolarization condition ([Ba^2+^]=10 mM), all maintaining consistent osmolarity by adjusting [Na^+^].

**Figure 4.**
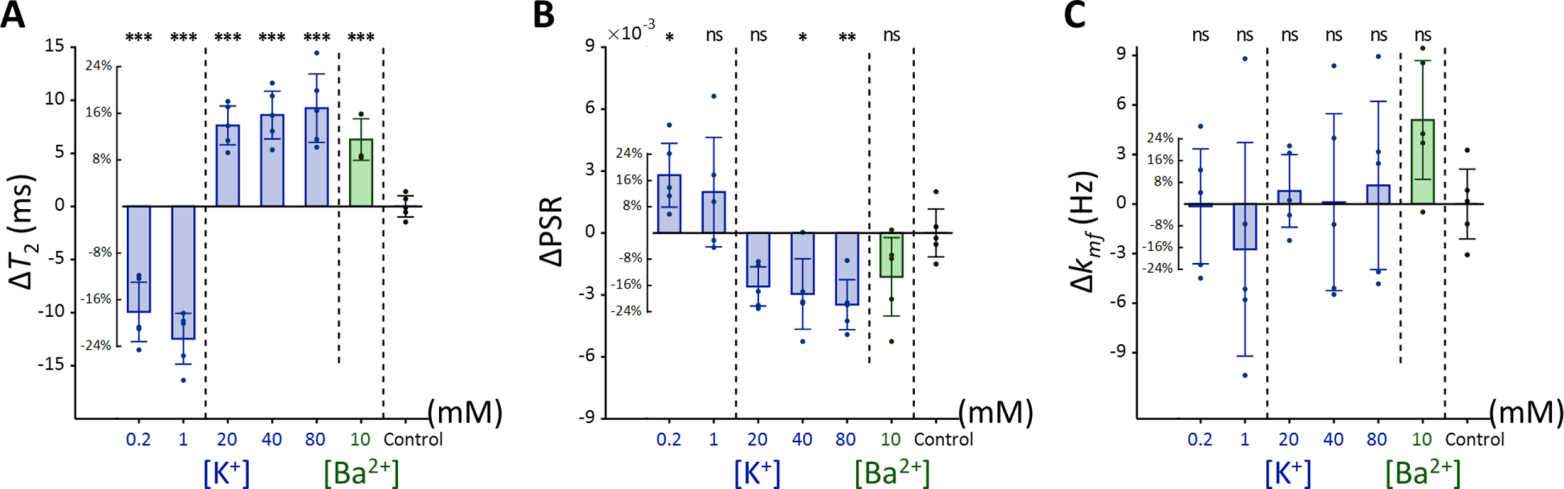
Changes in (**A**) *T*_2_, (**B**) PSR, and (**C**) *k_mf_* of Jurkat cells across experimental conditions: [K^+^]=0.2–80 mM (blue bar) and [Ba^2+^]=10 mM (green bar), compared to the control condition of [K^+^]=4.2 mM (n=5). Error bars denote standard deviation. Statistical significance of changes in MR parameters is marked with asterisks (ns: p>0.05, *: p<0.05, **: p<0.01, ***: p<0.001).

Each experimental condition was compared to the control using Dunnett’s test to account for multiple comparisons (Figure 4). As observed with SH-SY5Y cells, Jurkat cells showed significant positive changes in *T*_2_ and negative changes in PSR under increased [K^+^] conditions. For example, at [K^+^]=80 mM, *T*_2_ increased by 16.9% (Δ*T*_2_=9.26ms, p<0.0001), PSR decreased by 21.9% (ΔPSR = −0.00347, p<0.01). In contrast, during hyperpolarization at the lowest [K^+^]=0.2 mM, *T*_2_ decreased by 18.1% (Δ*T*_2_ = −9.93ms, p<0.0001), whereas PSR increased by 17.6% (ΔPSR = 0.00280, p<0.05). The depolarization induced by [Ba^2+^]=10 mM resulted in a similar pattern, with *T*_2_ increasing by 11.5% (Δ*T*_2_=6.3ms, p<0.0005), although the decrease in PSR was not significant (ΔPSR = −0.00212, p=0.211). Changes in *k_mf_* remained non-significant across all conditions (p>0.05). In summary, these findings indicate that detecting membrane potential changes induced by ionic solutions using MR parameters such as *T*_2_ and PSR is not specific to a single cell type, although the magnitude of these changes may differ between cell types.

### In vivo changes in *T*_2_ by membrane potential

**Figure 5.**
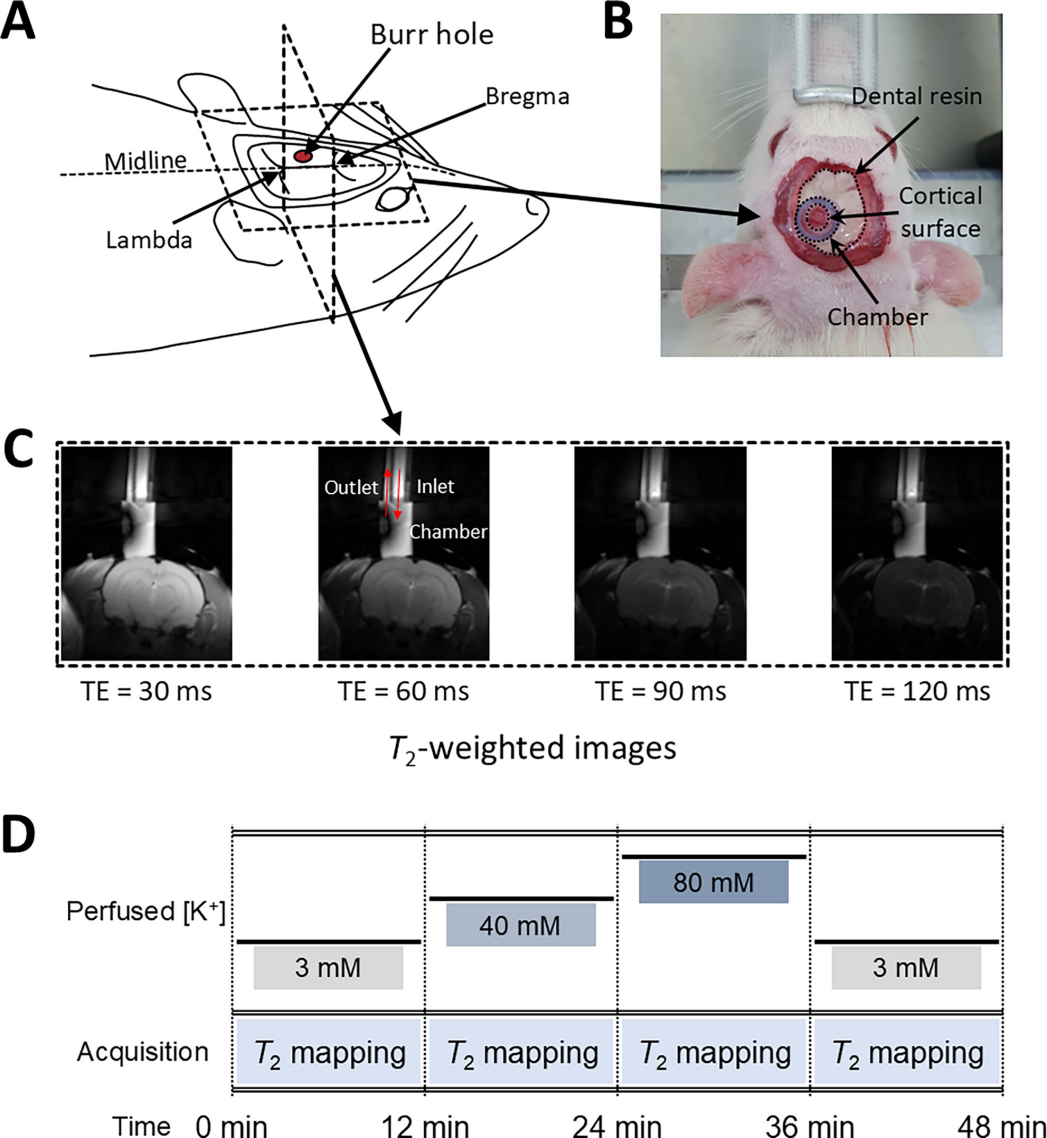
Experimental setup for in vivo manipulation of membrane potential. (**A**) A schematic diagram of the rat head post-craniotomy, showing the burr hole centered at 2.5 mm anterior and 2.0 mm lateral to the lambda. (**B**) Photograph of the rat head with a cylindrical chamber fixed over the burr hole, filled with artificial cerebrospinal fluid. (**C**) A representative series of *T*_2_-weighted MR images for *T*_2_ mapping. The chamber was connected to inlet and outlet perfusion tubes. (**D**) The experimental paradigm of the in vivo rat MR imaging. Four conditions were sequentially applied: control, depolarization, further depolarization, and recovery. Each condition lasted 12 min during which *T*_2_ mapping was conducted.

The relationship of *T*_2_ values and membrane potential modulated by [K^+^], observed in the aforementioned SH-SY5Y and Jurkat cell studies, was further explored in an in vivo rat model to validate these findings under physiological conditions. As depicted in Figure 5, a craniotomy was performed to expose a 3-mm-diameter region of the rat cerebral cortex, followed by perfusion with artificial cerebrospinal fluid (aCSF) to modulate the membrane potential. Hemodynamic responses were pharmacologically suppressed. MRI scans were performed using a 7T preclinical MRI system to measure *T*_2_ in the exposed cortical area. A total of seven rats were used in the experiment that involved modulation of [K^+^]. The experimental protocol included sequential application of four conditions, each lasting 12 min: a baseline condition at [K^+^]=3 mM, a depolarization condition at [K^+^]=40 mM, further depolarization at [K^+^]=80 mM, followed by a recovery condition using baseline aCSF. The recovery condition was applied to two of the seven rats. To distinguish the effect of aCSF perfusion on *T*_2_ from the effect of changes in membrane potential, a control experiment was also conducted using only baseline aCSF for the entire duration (48 min) with another group of five rats.

**Figure 6.**
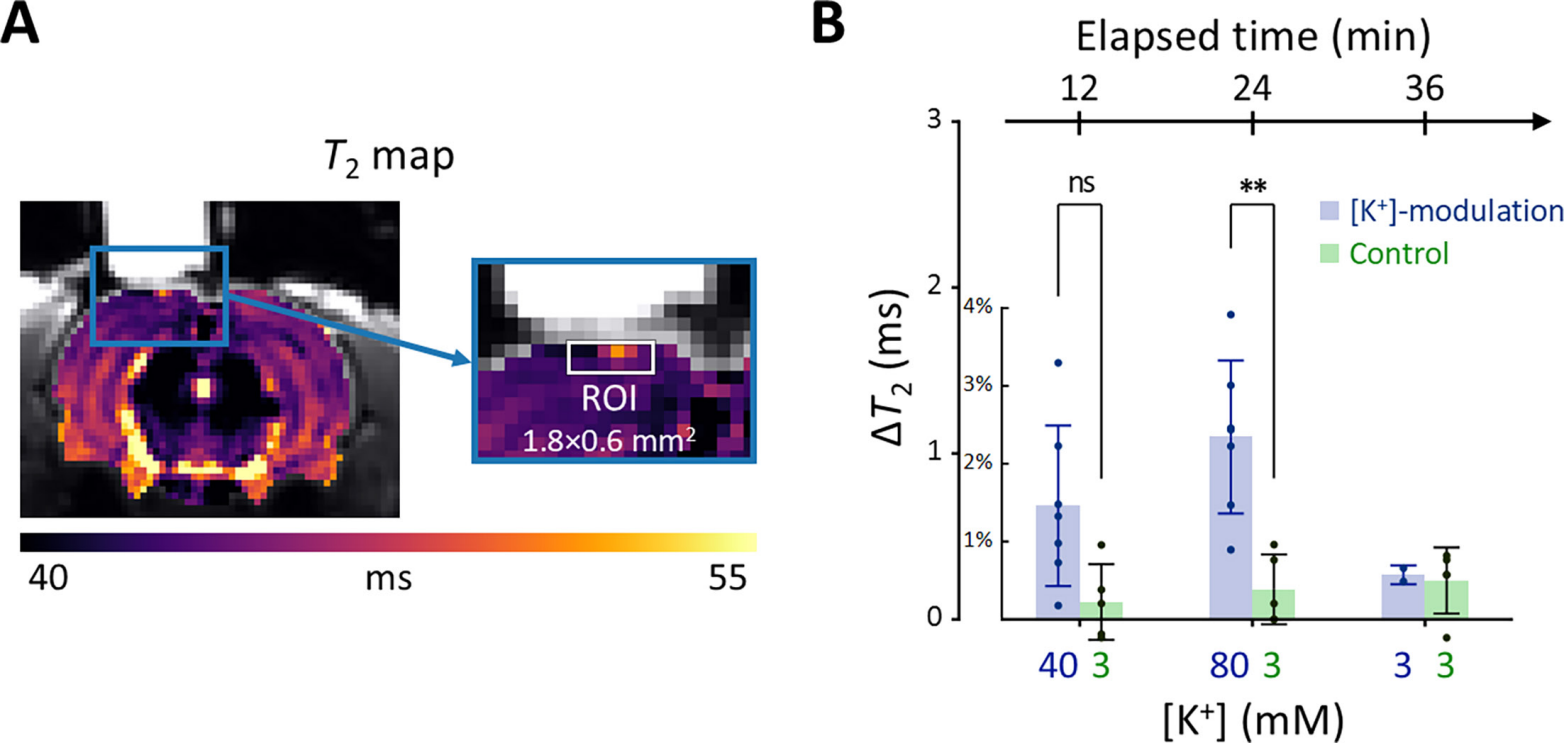
Results of the in vivo experiment results in rat models. (**A**) A representative T2 map from a single rat with an enlarged image depicting the ROI for estimating average T2 in the exposed cortical area, marked by a white rectangle (width = 1.8 mm, depth = 0.6 mm). (**B**) The changes in T2 values within the ROI is plotted against elapsed time from the initial conditions. [K^+^] in the perfused artificial cerebrospinal fluid are indicated on the bottom abscissa. Results from the [K^+^]-modulation experiments are shown with blue bars, and those from the control experiments are shown with green bars. Statistical significance of the T2 changes is marked with asterisks (ns: p > 0.05, *: p < 0.05, **: p< 0.01).

In Figure 6A, a representative *T*_2_ map is displayed with an enlarged image defining the ROI beneath the perfusion chamber (width = 1.8 mm, depth = 0.6 mm). The average *T*_2_ value within this ROI was estimated from the spatially averaged multi-echo spin-echo signal. A detailed analysis of the quality of these *T*_2_ maps is presented in Appendix 3. Changes in *T*_2_ value (Δ*T*_2_) were statistically analyzed using a linear mixed-effect model to account for inter-sample variability and repeated measurements. This model included Δ*T*_2_ as a dependent variable, elapsed time and its interaction with the experiment type (i.e. [K^+^]-modulation or control) as fixed effects, and a random effect for inter-sample variability.

Figure 6B shows the results of the statistical analysis. Δ*T*_2_ values were plotted against elapsed time for [K^+^]-modulation and control experiments. After 12 mins from the initial condition, *T*_2_ increased by 1.46% (Δ*T*_2_=0.684ms) in the [K^+^]-modulation experiment ([K^+^]=40 mM) and by 0.223% (Δ*T*_2_=0.104ms) in the control experiment ([K^+^]=3 mM); the Δ*T*_2_ difference (=0.580ms) between these experiments was not statistically significant (p=0.0711). After 24 min, *T*_2_ increased by 2.34% (Δ*T*_2_=1.10ms) in the [K^+^]-modulation experiment ([K^+^]=80 mM) and by 0.386% (Δ*T*_2_=0.181ms) in the control experiment ([K^+^]=3 mM), with a significant difference in Δ*T_2_* (=0.918ms) between them (p=0.00172). Due to the limited sample size in the recovery phase (n=2 out of 7 for [K^+^]-modulation), comparisons between experiments were not conducted for this recovery phase.

Our observations indicate that in vivo manipulation of membrane potential results in similar trends of *T*_2_ changes as those observed in vitro, albeit with a smaller magnitude in the rat cortex. This discrepancy may be attributed to several factors: [K^+^]-modulation affecting only a small portion of cells within the ROI; limited diffusion of aCSF through the leptomeninges; removal of excessive K^+^ through clearing mechanisms; and differences in cell types (see the Discussion for more details).

## Discussion

In this study, we demonstrated that MR parameters, specifically *T*_2_ relaxation time and pool size ratio (PSR), can detect responses to membrane potential changes modulated by ionic solutions. Our in vitro experiments with cultured cells were designed to exclude physiological factors such as hemodynamic responses and respiration. We observed that depolarization increases *T*_2_ and decreases PSR, while hyperpolarization has the opposite effect. In vivo, we pharmacologically suppressed hemodynamic responses to minimize their impact on *T*_2_ measurements. The trend of *T*_2_ dependence on membrane potential in vivo was consistent with in vitro findings. However, the magnitude of *T*_2_ changes in rat cortex was approximately one-ninth of that observed in SH-SY5Y cells in vitro at [K^+^]=80 mM.

Other studies have also reported MR-detectable changes in response to extracellular [K^+^] modulation. For instance, research on spreading depression, which was induced by significantly high [K^+^] (~1 M) applied topically to rat cortex, has revealed detectable changes in *T*_1_, *T*_2_, and magnetization transfer ratio changes (Stanisz et al., 2002) and spin-lock fMRI signals (Autio et al., 2014). Similarly, increased [K^+^] has been studied in vitro with brain slices, linking cell volume changes to proton density-weighted MR signal changes (Stroman et al., 2008). *T*_2_ mapping in Jurkat cells with increased [K^+^] conditions has also been investigated (Toi et al., 2022) and linked to cell volume change (Phi Van et al., 2024). Our study complements these findings by employing direct membrane potential measurement via patch clamp, testing additional ionic agents such as Ba^2+^, and demonstrating the phenomenon in vivo. Further reinforcement of these findings could be achieved by simultaneous recording of cell volume and other cellular characteristics to elucidate the underlying mechanisms more completely.

Interestingly, the MR responses in Jurkat cells differed from those in SH-SY5Y cells. While SH-SY5Y cells showed a near-linear dependence of MR parameters on log [K^+^], Jurkat cells displayed more step-like behavior. The reasons for this discrepancy remain unclear, but it suggests that the relationship between membrane potential and MR parameters may not strictly follow a simple log [K^+^] relationship.

Several factors may contribute to discrepancies between in vivo and in vitro results. For instance, the actual extracellular [K^+^] experienced by cells in the rat cortex may be lower than that of the perfused aCSF due to diffusion-limiting barriers such as the leptomeninges (Bradbury et al., 1972; Filippidis et al., 2012), even after the removal of the dura mater. Additionally, removal of excessive K^+^ by clearing mechanisms (Walz, 2000; O’Donnell, 2009) may further reduce the [K^+^] experienced by the cells. Partial volume effects and cell type differences may also contribute to the discrepancy.

From a biophysical perspective, the sensitivity of *T*_2_ and PSR to membrane potential likely arises from alterations in cell volume, hydration water, and bulk water. Depolarization or hyperpolarization can lead to cell swelling or shrinking (Lang et al., 1998; Fraser and Huang, 2004; Hoffmann et al., 2009), influencing the proportion of cellular contents within the imaging voxel and thereby affecting MR parameters. Notably, neuronal or glial cell swelling has been proposed as a possible mechanism underlying diffusion fMRI (Le Bihan et al., 2006; Le Bihan, 2012; Mangia et al., 2012) and previous MRI studies (Stroman et al., 2008; Phi Van et al., 2024). Although not as sensitive as *T*_2_, our *T*_1_ measurements (Appendix 2—figures 1–3) also exhibited similar trends in response to membrane potential changes. Moreover, hydration water, which has a significantly shorter *T*_2_ than bulk water due to slower re-orientational and diffusive motions (Mathur-De Vré, 1980), may contribute to the observed MR changes. In particular, the correlation between PSR and membrane potential indicates that depolarization decreases the density of hydration water on the cell membrane within a voxel, thereby reducing PSR and simultaneously increasing *T*_2_ due to a corresponding increase in free water, as well as cell swelling. Conversely, hyperpolarization may increase hydration water density, elevating PSR and lowering *T*_2_. These interpretations are supported by recent optical studies showing reduced membrane hydration water during depolarization (Didier et al., 2018).

Several challenges and considerations in this study warrants discussion. First, maintaining the desired environment (37 °C and 5% CO_2_) for in vitro cells during MRI scans is challenging. In addition, intracellular accumulation of Ba^2+^ in the Ba^2+^-induced depolarization experiment may affect cellular integrity. In this study, high cell viability (>97.6%) was confirmed using cell viability assays under experimental conditions (Appendix 4—figure 1). Second, differences in *T*_2_ value among the extracellular media may bias *T*_2_ measurements due to the partial volume effect. However, in this study, the differences in *T*_2_ among the extracellular media were found to be negligible compared to the observed *T*_2_ changes (Appendix 2—figure 4). Third, while our findings show that membrane potential-modulating ionic solutions can affect MR parameters, it is important to note that these changes do not measure the membrane potential itself. Fourth, other factors such as pH, energy depletion, or extracellular osmolarity may affect MR parameters by altering cell volume. To minimize the effects of these other contributors, we matched osmolarity across all conditions, provided sufficient glucose to prevent energy depletion, and regulated pH levels by buffering with HEPES. Additionally, to mitigate possible changes in intra/extracellular volume fraction changes caused by cell movements, potentially due to agitation, we centrifuged the cells and imaged the bottom portion of the cell pellet, where cell movement was restricted due to close packing. Fifth, in the in vivo study, we attempted to suppress hemodynamic responses through pharmacological means, using a combination of N_ω_-Nitro-L-arginine and nifedipine, both of which are known to inhibit hemodynamic responses in different ways (Dreier et al., 1995; Redmond et al., 2002), but their effects were not directly confirmed in this study. Future studies that simultaneously evaluate hemodynamic responses would strengthen our conclusions. Finally, our experimental paradigm was based on clamping the membrane potential at a specific level, thus measuring changes in *T*_2_ and PSR during static depolarization or hyperpolarization rather than dynamic changes such as those seen during action potentials. Future research could explore temporally varying membrane potential to evaluate the dynamic correlation between membrane potential and MR parameters with high temporal resolution MRI (Sundaram et al., 2010; Toi et al., 2022).

In summary, our study demonstrates that MR parameters such as *T*_2_ relaxation time can detect responses to membrane potential-modulating ionic solutions both in vitro and in vivo. This finding proposes a potential approach for noninvasively detecting changes in membrane potential using MRI.

## Materials and methods

**Key resources table:** 

Reagent type (species) or resource	Designation	Source or reference	Identifiers	Additional information
Strain, strain background (*Rattus norvegicus*)	Wistar	Orient Bio	Cat #: CrlOri:WI; RRID:RGD_13508588	
Cell line (*Homo sapiens*)	SH-SY5Y	American Type Cell Collection	Cat #: CRL-2266; RRID:CVCL_0019	
Cell line (*Homo sapiens*)	Jurkat E6.1	American Type Cell Collection	Cat #: TIB-152; RRID:CVCL_0367	

### In vitro cell culture

Two human cell lines were utilized for the experiments: SH-SY5Y, an immortalized neuroblastoma line, and Jurkat, a leukemia cell line. Both cell lines were sourced from the American Type Culture Collection (ATCC), where their identities were authenticated by STR analysis and confirmed to be negative for mycoplasma contamination, as documented in the certificate of analysis provided at the time of purchase. The SH-SY5Y cells were cultured in DMEM/F12 medium supplemented with 10% (v/v) fetal bovine serum (FBS) and 100 U/ml penicillin/streptomycin. The cells were maintained at a constant temperature of 37 °C in a humidified atmosphere containing 5% CO_2_. Similarly, the Jurkat cells were cultured in RPMI-1640 medium, also supplemented with 10% (v/v) FBS and 100 U/ml penicillin/streptomycin, under the same conditions of temperature and CO_2_ concentration.

### In vitro manipulation of membrane potential with extracellular media

The baseline extracellular medium was prepared with the following components: KCl = 4.2 mM; NaCl = 145.8 mM; HEPES = 20 mM; glucose = 4.5 g/l; EGTA = 10 µM; pH = 7.2. This baseline medium was considered a control condition for various extracellular media used to adjust membrane potential. Two extracellular media with low K^+^ concentrations (KCl = 0.2 and 1 mM) were prepared to hyperpolarize the membrane potential. Three extracellular media with high K^+^ concentrations (KCl = 20, 40, and 80 mM) were prepared to depolarize the membrane potential. A Ba^2+^ medium containing 10 mM BaCl_2_ was also prepared to depolarize the membrane potential in a different way, i.e., as a K^+^ channel blocker. These seven extracellular media were used for both MR imaging and patch clamp recording in vitro. Sodium ion concentrations ([Na^+^]) in all media were controlled to match the osmolarity with the baseline medium. The composition of all extracellular media is detailed in Appendix 1—table 3.

### Preparation of cells for in vitro MR measurement

SH-SY5Y cells were dissociated from their culture plates using 0.5 mM EDTA solution, then concentrated into a pellet (~70 µl) by centrifugation at 250×g for two minutes. The pellet was resuspended in the culture medium and divided evenly into seven aliquots. Each cell suspension was centrifuged and resuspended in each of the seven different media specified in Appendix 1—table 3. Centrifugation and resuspension were repeated three more times to completely clear out the culture medium. Each cell suspension with a different extracellular medium was then loaded into two wells on the same column of an acrylic container with 14 wells (matrix = 2 × 7) and centrifuged again to concentrate into pellets (Figure 1). The acrylic container was purposely formed into a spherical segment to enhance the homogeneity of the static magnetic field (Lee et al., 2020). The preparation of Jurkat cell samples was the same as for the SH-SY5Y cell samples. The preparation required 40–60 min, followed by an incubation period of 20–30 min.

### In vitro MRI experiment

In vitro MRI experiments were performed on a 9.4T MRI system (BioSpec 94/30 USR, Bruker BioSpin) at room temperature. A volume coil with an inner diameter of 86 mm was utilized for both radiofrequency (RF) pulse transmission and signal reception. Within the acrylic container, two wells on the same column (matrix = 2 × 7) contained identical cell pellets with the same extracellular media. MRI signals from seven different cell samples in the horizontal direction were separated by one-dimensional frequency encoding along that direction (Figure 1). The MRI pulse sequence employed for mapping the *T*_2_ value was a single-echo spin-echo (SESE) sequence with 50 variable echo times (TE) spaced between 9.5 and 290.5ms on a logarithmic scale. For mapping the MT parameters, an inversion recovery multi-echo spin-echo (IR-MESE) sequence was used. The inversion times (TI) for the IR-MESE sequence were optimized using the theory of Cramér-Rao lower bounds (Li et al., 2010). The optimized TIs ranged from 4 to 10,079.4ms. After each TI, 16 spin-echo trains were acquired with an echo spacing of 9.5ms. Total scan time for both sequences was 23 min. Experiments were repeated 15 times for SH-SY5Y cells and 7 times for Jurkat cells, replacing cells in each repetition. Other scan parameters are detailed in Appendix 1—table 1.

### Animals

Male Wistar rats aged 8 weeks (250–300 g, Orient Bio) were used for MRI experiments after undergoing a craniotomy. All animal experiments were approved by the Institutional Animal Care and Use Committee at the National Cancer Center Korea (NCC-22–740). The rats were housed in ventilated cages under a 12 hr/12 hr light/dark cycle and provided with ad libitum access to food and water.

### In vivo manipulation of membrane potential with artificial cerebrospinal fluid (aCSF)

The membrane potential of the exposed cortex was manipulated by directly perfusing the region of interest of the cerebral cortex with aCSF after a craniotomy. The baseline aCSF was prepared with the following components: KCl = 3 mM; NaCl = 135 mM; MgCl_2_ = 3 mM; HEPES = 20 mM; glucose = 4.5 g/l; EGTA = 2 mM; N_ω_-Nitro-L-arginine=1 mM; Nifedipine = 0.1 mM; pH = 7.4. Hemodynamic effects were pharmacologically suppressed using N_ω_-Nitro-L-arginine, Nifedipine, and EGTA. N_ω_-Nitro-L-arginine suppresses depolarization-induced hemodynamic response by blocking the synthesis of nitric oxide, which acts as a vasodilator (Dreier et al., 1995). Nifedipine blocks voltage-sensitive Ca^2+^ channels, and EGTA chelates free Ca^2+^ to inhibit the hemodynamic response (Redmond et al., 2002). To induce depolarization, aCSF with high [K^+^] (KCl = 40 and 80 mM) was prepared. The osmolarity of the aCSF was matched with the concentration of NaCl.

### Rat surgery

A craniotomy was performed on a Wistar rat. The experimental setup after a surgical procedure is illustrated in Figure 5. The surgery was performed following an established protocol (Mostany and Portera-Cailliau, 2008) alike to that used in other MRI studies (Stanisz et al., 2002; Autio et al., 2014). Anesthesia was induced with 3% isoflurane in O_2_ and maintained with 2–3% isoflurane during the surgical procedure. Body temperature was maintained at 36.5–37.5°C with an infrared lamp. The head was fixed with a small animal stereotaxic frame. The hair on the scalp was shaved with a veterinary clipper. The skin and periosteum over the skull were removed using a scalpel and surgical scissors. A 3.0-mm-diameter burr hole was opened using a dental drill with its center at the coordinates of 2.5 mm anterior and 2.0 mm lateral to the lambda. Then, a cylindrical chamber was implanted upon the burr hole with cyanoacrylate glue and dental composite resin. The chamber was filled with the baseline aCSF and connected to inlet and outlet perfusion tubes. The exposed cerebral cortex inside the chamber was perfused with the baseline aCSF at a flow rate of 0.6 ml/min using peristaltic pumps.

### In vivo MRI experiment

The rat with a cranial chamber installed on the cortical surface was placed on a 7T MRI system (BioSpec 70/20 USR, Bruker BioSpin), fixed in a customized cradle with two ear-bars and a bite-bar. A customized surface coil (rectangular, 35 mm × 20 mm) was used for RF pulse transmission and signal reception. Body temperature was maintained at 36.5–37.5°C using a warm air blower. Anesthesia was maintained with 2% isoflurane in O_2_ (0.6 l/min). Respiration rate and body temperature were monitored throughout the MRI experiment. MR images were acquired in a 2 mm coronal slice through the center of the burr hole (Figure 5), using a multi-echo spin-echo (MESE) sequence with 20 TEs (7.5–150ms). Other scan parameters are detailed in Appendix 1—table 2.

A total of seven rats were subjected to four sequential experimental conditions, as depicted in Figure 5. First, the exposed cerebral cortex was perfused with baseline aCSF ([K^+^]=3 mM). Second, as a depolarizing condition, the perfusion media was switched to depolarizing aCSF of [K^+^]=40 mM. Third, membrane potential was further depolarized by perfusion with aCSF of [K^+^]=80 mM. Finally, as a recovery condition, the perfused aCSF was changed back to baseline aCSF. During each condition, MR images were acquired with MESE sequences for 12 min. Two rats underwent the whole four conditions, while five other rats did not undergo the recovery condition. As a control experiment, another set of rats (n=5) underwent perfusion with the baseline aCSF for the same duration (48 min) as the previous experiment, and MR images were acquired with four MESE sequences, each for 12 min. Throughout the experiment, the perfusion rate was maintained at 0.6 ml/min.

### Quantification of MR parameters

For the in vivo MR images acquired with a MESE sequence, echo trains were matched with a simulated dictionary of decay curves of multi-echo spin-echo signals created with the stimulated echo and slice profile correction (McPhee and Wilman, 2017) to estimate *T*_2_ values. For the in vitro one-dimensional MR images acquired with the SESE sequence, signals were fitted to a mono-exponential function to estimate *T*_2_ values. For the in vitro one-dimensional MR images acquired with the IR-MESE sequence, signals were fitted to a bi-exponential function (Edzes and Samulski, 1977; Gochberg and Gore, 2003) to estimate MT parameters. 16 spin echoes acquired after each TI were averaged to improve SNR. The MT parameters such as PSR and *k_mf_* were derived from this fitting process.

A 1ms hard inversion pulse selectively inverted the magnetization of free water protons, leading to cross-relaxation between the longitudinal magnetization of free water protons (*M_z_*_,*f*_) and macromolecular protons (*M_z_*_,*m*_). This interaction resulted in a bi-exponential magnetization recovery characterized by a fast longitudinal relaxation rate $R_{1}^{+}$ and a slow relaxation rate $R_{1}^{-}$ (=1 */*_T1_), with the latter corresponding to the conventional spin-lattice relaxation rate (Gochberg and Gore, 2003; Xu et al., 2014):

(4) $$\displaystyle  \frac{M_{z,f}(t)}{M_{\infty,f}} = b_f^+ \exp ({-R_1^+ t}) + b_f^- \exp ({-R_1^- t}) + 1$$

where *M*_∞,*f*_ denotes the equilibrium magnetization of free water protons, and $b_{f}^{+}$ and $b_{f}^{-}\,$ denote the amplitudes for the exponential terms associated with $R_{1}^{+}$ and $R_{1}^{-}$ ,respectively. By fitting this bi-exponential model to the inversion recovery signals using least squares, estimates of $R_{1}^{+}$, $R_{1}^{-}$, $b_{f}^{+}$, and $b_{f}^{-}$ were obtained. These parameters are related to the MT parameters, PSR and *k_mf_*, by the following equations:

(5) $$\displaystyle  2R_{1}^{\pm }=R_{1,f}+R_{1,m}+k_{fm}+k_{mf}\pm \sqrt{\left (R_{1,f}-R_{1,m}+k_{fm}-k_{mf}\right)^{2}+4k_{fm}k_{mf}}$$

(6) $$\displaystyle  b_{f}^{\pm }=\pm \frac{\left (R_{1,f}-R_{1}^{\mp }\right)\left (\frac{M_{z,f}\left (0\right)}{M_{\mathrm{\infty },f}}-1\right)+k_{fm}\left (\frac{M_{z,f}\left (0\right)}{M_{\infty ,f}}-\frac{M_{z,m}\left (0\right)}{M_{\infty ,m}}\right)}{R_{1}^{+}-R_{1}^{-}}$$

(7) $$\displaystyle  PSR=k_{fm}/k_{mf}$$

where *R*_1,*f*_ and *R*_1,*m*_ denote the longitudinal relaxation rates of free water and macromolecular protons, respectively, in the absence of cross-relaxation. *k_fm_* and *k_mf_* denote the magnetization transfer rates from free water to macromolecules and vice versa, respectively, *M_z_*_,*m*_ (0) denotes the longitudinal magnetization of macromolecular protons immediately after the inversion pulse. *M*_∞,*m*_ denotes the equilibrium magnetization of macromolecular protons. According to previous studies (Gochberg and Gore, 2003; Gochberg et al., 1999), *M_z_*_,*m*_ (0)/*M_∞_*_,*m*_ can be determined numerically by the Bloch equations. Assuming *R*_1,*f*_ = *R*_1,*m*_ (Li et al., 2010; Cabana et al., 2015), the equations [Equations 5–7] can be simplified to explicitly calculate PSR and *k_mf_* .

(8) $$\displaystyle  \text{PSR} = \frac{b_f^+}{b_f^- - \frac{M_{z,m}(0)}{M_{\infty,m}} + 1}$$

(9) $$\displaystyle  k_{mf} = \frac{R_1^+ - R_1^-}{1 + \text{PSR}}$$

### Patch clamp recordings

The membrane potential of SH-SY5Y cells was recorded at room temperature using the whole-cell mode of the patch clamp technique (Hamill et al., 1981). The bath solution was the same as the extracellular medium used in the MRI experiment and was constantly perfused at a flow rate of 2 ml/min. The composition of the pipette solution was as follows: KCl = 140 mM; NaCl = 5 mM; MgCl2=3 mM; HEPES = 10 mM; Mg-ATP=1 mM; Na-GTP=0.5 mM. The pH of the pipette solution was adjusted to 7.4 using KOH. Calcium ions (Ca^2+^) were not included in the pipette solution to minimize Ca^2+^-dependent currents. The resistance of the electrode was 3–5 MΩ with the internal solution filled. Recordings were performed with a patch amplifier (Axopatch-1D; Axon Instruments) and a current clamp was also used to monitor the membrane potential. The experiment was repeated three times, with cells replaced at each repetition.

## Figures and Tables

**Appendix 2—figure 1.**
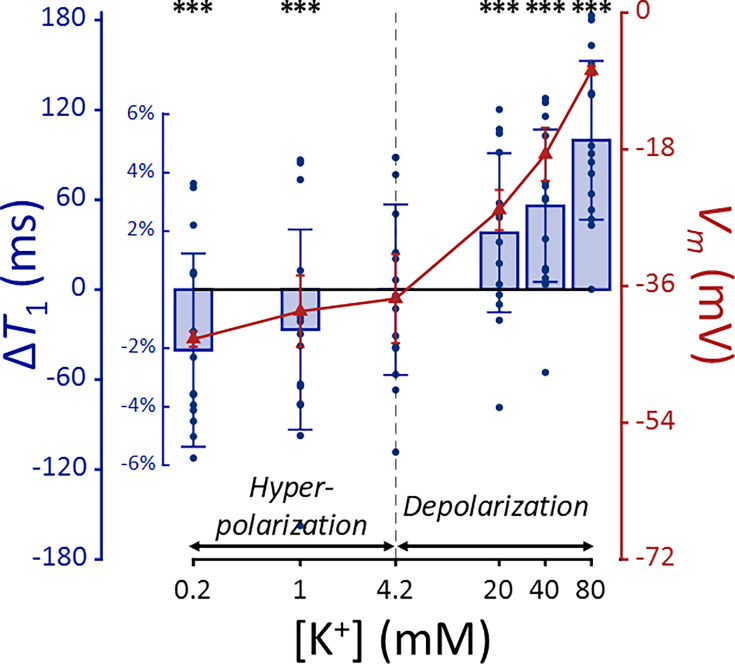
T_1_ and membrane potential (V_m_) of SH-SY5Y cells versus extracellular K^+^ concentrations ([K^+^]). Changes in T_1_ are displayed with blue bars (n = 15). Membrane potentials are plotted with red triangles (n = 3). The abscissa is in logarithmic scale. Error bars denote standard deviation. Statistical significance of changes in T1 is marked with asterisks (***: p < 0.001).

**Appendix 2—figure 2.**
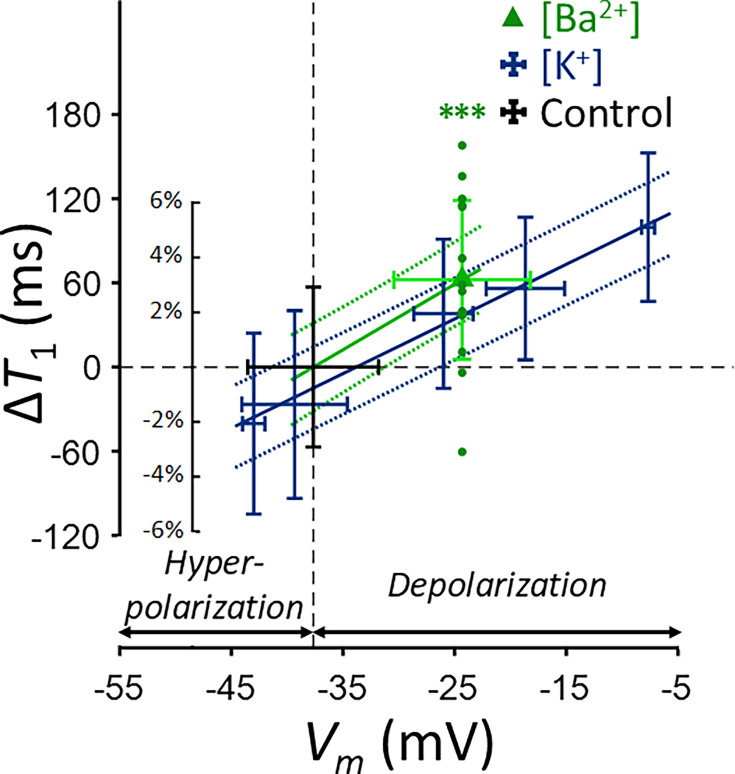
Changes in T_1_ of SH-SY5Y cells across experimental conditions: [K^+^] = 0.2–80 mM (blue cross) and [Ba^2+^] = 10 mM (green triangle), compared to the control condition (black cross). Data from fifteen experiments (n = 15) are displayed. Linear regression lines for [K^+^] data (blue solid line) and [Ba^2+^] data (green solid line) are drawn along with 95% confidence intervals. Error bars denote standard deviation. Statistical significance of changes in T_1_ with [Ba^2+^] = 10 mM is marked with asterisks (***: p < 0.001).

**Appendix 2—figure 3.**
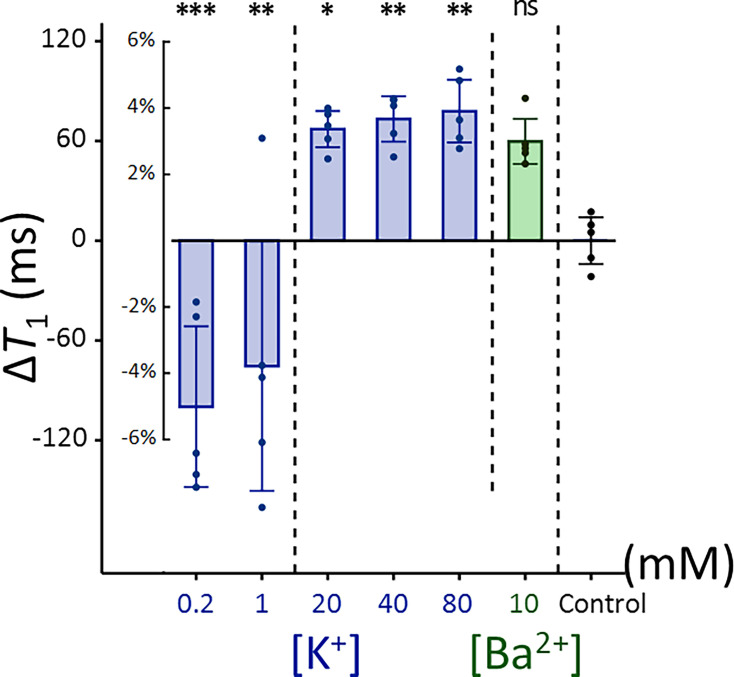
Changes in T_1_ of Jurkat cells across experimental conditions: [K^+^] = 0.2–80 mM (blue bar) and [Ba^2+^] = 10 mM (green bar), compared to the control condition of [K^+^] = 4.2 mM (n = 5). Error bars denote standard deviation. Statistical significance of changes in T_1_ is marked with asterisks (ns: p > 0.05, *: p < 0.05, **: p < 0.01, ***: p < 0.001).

**Appendix 2—figure 4.**
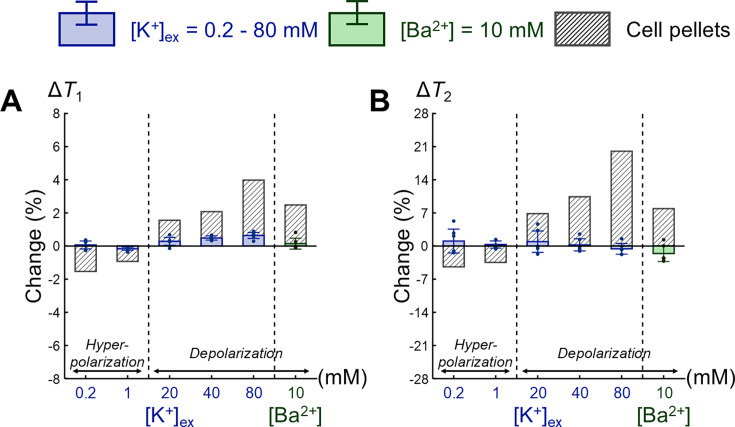
Comparison of changes in relaxation times between extracellular media and SH-SY5Y cell pellets. Changes in (A) T_1_ and (B) T_2_ are expressed as percentage changes relative to the control condition of [K^+^] = 4.2 mM. The results for extracellular media are displayed as blue bars for the extracellular K^+^ concentrations ([K^+^]) and green bars for the Ba^2+^ concentration ([Ba^2+^]) (n = 6). For comparison, the results for SH-SY5Y cell pellets corresponding to each extracellular medium are plotted. Error bars denote standard deviation.

**Appendix 3—figure 1.**
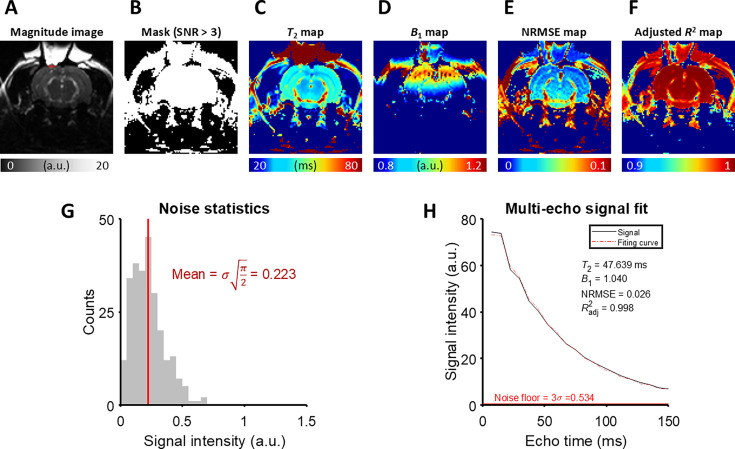
The detailed analysis of T_2_ fitting on an example MESE image from the in vivo rat experiment. (**A**) A magnitude image at the last echo time. The ROI is marked with a red rectangle. (**B**) A mask generated by thresholding the magnitude image with SNR > 3. (**C**) T_2_ map, (**D**) B_1_ map, (**E**) NRMSE map, and (**F**) Adjusted R^2^ map produced by the fitting procedure. (**G**) The noise statistics of the background of the magnitude image. (**H**) The magnitude signal averaged over the ROI and its fitting curve.

**Appendix 4—figure 1.**
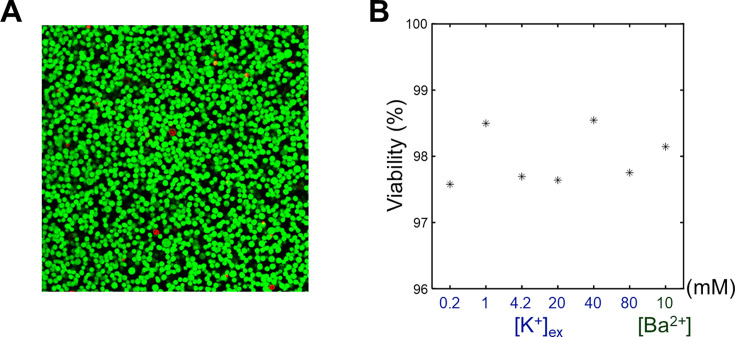
The viability assay of SH-SY5Y cells. (**A**) A representative confocal microscopy image of an SH-SY5Y pellet. [K^+^] of extracellular medium was 4.2 mM. Live cells (green) were stained with calcein-AM, and dead cells (red) were stained with EthD-1. (**B**) The viabilities of SH-SY5Y cells versus the extracellular K^+^ concentrations ([K^+^]) and Ba^2+^ concentration ([Ba^2+^]).

## Data Availability

The source data used to generate the figures are included in Source data 1.

## Additional files

MDAR checklist

Source data 1. This source data was used for generating the figures in this article.

## Appendix 1

This appendix provides detailed information on the experimental parameters and conditions used in both in vitro and in vivo MRI studies, as well as the composition of extracellular media applied to modulate cellular membrane potential.

**Appendix 1—table 1.** The scan parameters of the sequences used in the in vitro MR experiments. The cell samples were scanned with two types of sequences, single-echo spin-echo (SESE) and inversion recovery multi-echo spin-echo (IR-MESE) sequences, on a 9.4 T MRI. T_2_ was estimated from the SESE sequence. T_1_, PSR, and k_mf_ were estimated from the IR-MESE sequence, and its inversion times (TIs) were optimized using the theory of Cramér-Rao low bounds: TIs = 4, 4, 4, 4, 4, 4, 4, 4, 17.91, 18.18, 18.18, 18.2, 18.2, 18.21, 18.21, 18.24, 18.24, 18.24, 18.24, 18.24, 18.31, 18.31, 18.32, 18.46, 55.18, 55.26, 55.53, 55.98, 163.5, 164.83, 164.92, 164.93, 164.96, 165.25, 165.25, 165.62, 197.68, 1976.77, 2280.95, and 10076.4 ms. The recovery time or repetition time was set long enough to ensure full relaxation of nuclear magnetization.

Sequence type	SESE	IR-MESE
Recovery time (ms)	N/A	15000
Repetition time (ms)	15000	N/A
Inversion time (ms)	N/A	4–10,079.4 (40 steps)
Echo time (ms)	9.5–290.5 (linear, 50 steps)	9.5–152 (linear, 16 steps)
Resolution (mm)	0.5	
Slice thickness (mm)	1	
Estimated parameters	T_2_	T_1_, PSR, and k_mf_

**Appendix 1—table 2.** The scan parameters of the sequence used in the in vivo rat MR experiments. The rats were scanned with a multi-echo spin-echo (MESE) sequence on a 7 T MRI.

Sequence type	MESE
Repetition time (ms)	1000
Inversion time (ms)	N/A
Echo time (ms)	7.5–150 (linear, 20 steps)
Resolution (mm^2^)	0.3×0.3
FOV (mm^2^)	28.8×28.8
Slice thickness (mm)	2
Estimated parameters	T_2_

**Appendix 1—table 3.** The composition of the extracellular media used to modulate membrane potential in vitro. The sodium chloride concentrations were adjusted to maintain the same osmolarity across all media. Besides the inorganic salts listed in this table, all media commonly contained HEPES = 20 mM; glucose = 4.5 g/l; EGTA = 10 μM; pH = 7.2.

Medium type	KCl (mM)	BaCl_2_ (mM)	NaCl (mM)
Baseline	4.2		145.8
Low [K^+^]	0.2		149.8
	1		149
High [K^+^]	20		130
	40		110
	80		70
[Ba^2+^]	4.2	10	130.8

## Appendix 2

### Membrane potential-induced changes in *T*_1_: SH-SY5Y cells

The changes of *T*_1_ in SH-SY5Y cells when the membrane potential (*V_m_*) was modulated by varying [K^+^] = 0.2–80 mM or adding [Ba^2+^] = 10 mM are shown in Appendix 2—figures 1 and 2. A linear mixed-effect model was employed to analyze the effect of changes in *V_m_* (Δ*V_m_*) on *T*_1_. This model included *T*_1_ as a dependent variable, Δ*V_m_* and its interaction with a group variable (indicating whether Δ*V_m_* is induced by [Ba^2+^] or [K^+^]) as fixed effects, and cell batch as a random effect. The analysis revealed no significant interaction term (*p* = 0.188), showing that changes in *T*_1_ do not depend on the specific method of membrane potential modulation. The model yielded the following relationship:

(A2.1) $$\displaystyle  T_1\ (\text{ms}) = 1.94 \times 10^3 \left(1 + 0.00197\, \Delta V_m\right) $$

The effect Δ*V_m_* on *T*_1_ was significant (p < 0.0001), indicating an increase in *T*_1_ during depolarization and a decrease during hyperpolarization. This trend is the same with *T*_2_ regarding membrane potential, while the sensitivity is 3.5 times smaller compared to *T*_2_. Subsequent post-hoc analysis compared each experimental condition to the control using Dunnett’s test to account for multiple comparisons, showing significant changes in *T*_1_ across all conditions tested (p < 0.0001).

### Membrane potential-induced changes in *T*_1_: Jurkat cells

The changes in *T*_1_ were also evaluated using a different cell line, the Jurkat, under the same experimental conditions applied to SH-SY5Y cells. Each experimental condition was compared to the control using Dunnett’s test to account for multiple comparisons (Appendix 2—figure 3). As the same with observation in SH-SY5Y cells, Jurkat cells showed positive changes in *T*_1_ during hyperpolarization and negative changes during depolarization, indicating that the detectability of membrane potential changes by *T*_1_ does not appear specific to cell type.

### Quantification of *T*_1_ and *T*_2_ of extracellular media

To estimate the *T*_1_ and *T*_2_ of extracellular media specified in Appendix 1—table 3, 200 μl of each media sample was loaded into two wells on the same column of the acrylic container with 14 wells (Figure 1A) and placed in a 9.4 T preclinical MRI system (BioSpec 94/30 USR, Bruker BioSpin). *T*_1_ and *T*_2_ were measured using an adiabatic inversion recovery multi-echo spin-echo sequence. 20 variable inversion times (TI) were spaced between 600 and 15,000 ms on a logarithmic scale. After each TI, 50 multi-echo spin-echo trains were acquired with an echo spacing of 9.5 ms (echo time (TE) = 9.5 to 475 ms). For the *T*_1_ estimation, the 50 multi-echo spin-echo signals acquired after each TI were averaged and fitted to a mono-exponential function:

(A2.2) $$\displaystyle  S(t) = A + B \exp\left(-\frac{t}{T_1}\right)$$

where *S*(*t*) denotes the averaged signal after an inversion time of *t*, and A and B denote the coefficients of the fitting. For *T*_2_ estimation, the 20 spin-echo signals at the same TE but different 20 TIs were averaged. These averaged 50 multi-echo spin-echo trains were then matched with a simulated dictionary of decay curves. This dictionary was constructed from simulation of multi-echo spin-echo signals, incorporating corrections for stimulated echoes and slice profile effects (McPhee and Wilman, 2017).

## Appendix 3

### Quantitative assessment of in vivo T_2_ mapping

In this study, in vivo *T*_2_ mapping was conducted using a multi-echo spin-echo (MESE) sequence. MESE signals exhibit nonexponential decay patterns due to stimulated echoes and imperfect slice profiles (Hennig, 1991). To correct for these effects, the MESE signals were fitted to a simulated dictionary of MESE signals, which was created using the extended phase graph (EPG) method (McPhee and Wilman, 2017). This dictionary included 360,000 MESE signal curves, spanning *T*_2_ ranges from 20 to 200 ms and *B*_1_ ranges from 0.5 to 1.5.

Appendix 3—figure 1 illustrates the procedure to assess the SNR and fitting quality for *T*_2_ mapping using an example MESE image from the in vivo rat experiment. First, the background noise of the MESE image was calculated. When a complex MR image contains Gaussian noise with a standard deviation of *σ*, the resulting magnitude image will exhibit Rician noise. This can be approximated as Gaussian noise if the SNR (= *A*/*σ*) is greater than 3, where *A* is the magnitude of signal in the absence of noise (Gudbjartsson and Patz, 1995). Rician noise was sampled from 64 voxels in the background of the magnitude image and the noise level *σ* was calculated from the average of the sampled Rician noise *M* using the formulaxels in the background of the magnitude (Gudbjartsson and Patz, 1995):

(A3.1) $$\displaystyle  M = \sigma \sqrt{\frac{\pi}{2}}$$

Subsequently, a mask was created by thresholding the magnitude image at SNR > 3. *T*_2_ mapping was performed by fitting the MESE signal to the simulated dictionary across the masked region. The fit quality was assessed using the normalized root mean squared error (NRMSE) and adjusted *R*^2^, calculated as follows:

(A3.2) $$\displaystyle  \text{NRMSE} = \frac{\sum (y_i - f_i)^2}{\sum y_i^2}$$

(A3.3) $$\displaystyle  \text{Adjusted } R^2 = 1 - \frac{n - 1}{n - p - 1} \frac{\sum (y_i - f_i)^2}{\sum (y_i - \bar{y})^2} $$

where is the mean of the signals, *y_i_* is the *i*^th^ signal, *f_i_* is the *i*^th^ fitted value, *n* is the sample size (number of echoes), and *p* is the number of variables.

Finally, the magnitude signal in the ROI beneath the perfusion chamber (width = 1.8 mm, depth = 0.6 mm) was analyzed. After averaging the magnitude signals over the ROI, it is confirmed that the signal is well above the noise floor (3*σ*). After performing *T*_2_ fitting, the fit quality was examined with NRMSE and adjusted *R*^2^.

This analysis was conducted across all seven in vivo rat models. Source data 1 contains the fitted *T*_2_ and *B*_1_ value, NRMSE and adjusted *R*^2^ value, the signal intensity of the last echo in the ROI, and the noise level *σ*. Notably, the maximum NRMSE was 0.043 and the minimum adjusted *R*^2^ was 0.995, indicating a high degree of alignment between the observed magnitude signals and the fitting curves.

## Appendix 4

### Viability assay of SH-SY5Y cells with live/dead staining

SH-SY5Y cells were cultured, harvested, and centrifuged to obtain a cell pellet. This cell pellet was resuspended in the culture medium and divided into seven equal aliquots. Each aliquot was centrifuged and resuspended three times using one of the seven different media specified in Appendix 1—table 3. For the viability assay, staining media were prepared by adding 2 μM calcein-AM and 4 μM Ethidium homodimer-1 to each of the seven media. The cell suspensions were then centrifuged and resuspended again using the staining media. Following this, each cell suspension was transferred to separate wells in a chambered coverglass and incubated at room temperature for 20 minutes. After another round of centrifugation, cell pellets were obtained and analyzed using a confocal microscope (Leica TCS SP8 STED, Leica Microsystems). Cell viability was assessed based on green fluorescence indicating live cells and red fluorescence indicating dead cells, under excitation wavelengths of 488 and 543 nm, respectively. Live and dead cells were manually counted with QuPath (Bankhead et al., 2017), and the cell viability was calculated as the ratio of live cells to total cells.

## References

[bib1] Abdul Kadir L, Stacey M, Barrett-Jolley R (2018). Emerging roles of the membrane potential: action beyond the action potential. Frontiers in Physiology.

[bib2] Autio JA, Shatillo A, Giniatullin R, Gröhn OH (2014). Parenchymal spin-lock fmri signals associated with cortical spreading depression. Journal of Cerebral Blood Flow & Metabolism.

[bib3] Bai R, Stewart CV, Plenz D, Basser PJ (2016). Assessing the sensitivity of diffusion MRI to detect neuronal activity directly. PNAS.

[bib4] Bandettini PA, Petridou N, Bodurka J (2005). Direct detection of neuronal activity with MRI: fantasy, possibility, or reality?. Applied Magnetic Resonance.

[bib5] Bankhead P, Loughrey MB, Fernández JA, Dombrowski Y, McArt DG, Dunne PD, McQuaid S, Gray RT, Murray LJ, Coleman HG, James JA, Salto-Tellez M, Hamilton PW (2017). QuPath: Open source software for digital pathology image analysis. Scientific Reports.

[bib6] Berger H (1929). Über das elektrenkephalogramm des menschen. Archiv Für Psychiatrie Und Nervenkrankheiten.

[bib7] Bradbury MWB, Segal MB, Wilson J (1972). Transport of potassium at the blood—brain barrier. The Journal of Physiology.

[bib8] Cabana JF, Gu Y, Boudreau M, Levesque IR, Atchia Y, Sled JG, Narayanan S, Arnold DL, Pike GB, Cohen-Adad J (2015). Quantitative magnetization transfer imaging made easy with qMTLab: Software for data simulation, analysis, and visualization. Concept Magn Reson A.

[bib9] Chow LS, Cook GG, Whitby E, Paley MNJ (2006). Investigating direct detection of axon firing in the adult human optic nerve using MRI. NeuroImage.

[bib10] Chu R, de Zwart JA, van Gelderen P, Fukunaga M, Kellman P, Holroyd T, Duyn JH (2004). Hunting for neuronal currents: absence of rapid MRI signal changes during visual-evoked response. NeuroImage.

[bib11] Cohen D (1968). Magnetoencephalography: evidence of magnetic fields produced by alpha-rhythm currents. Science.

[bib12] Didier MEP, Tarun OB, Jourdain P, Magistretti P, Roke S (2018). Membrane water for probing neuronal membrane potentials and ionic fluxes at the single cell level. Nature Communications.

[bib13] Dreier JP, Körner K, Görner A, Lindauer U, Weih M, Villringer A, Dirnagl U (1995). Nitric oxide modulates the cbf response to increased extracellular potassium. Journal of Cerebral Blood Flow & Metabolism.

[bib14] Edzes HT, Samulski ET (1977). Cross relaxation and spin diffusion in the proton NMR or hydrated collagen. Nature.

[bib15] Filippidis AS, Zarogiannis SG, Ioannou M, Gourgoulianis K, Molyvdas PA, Hatzoglou C (2012). Permeability of the arachnoid and pia mater: The role of ion channels in the leptomeningeal physiology. Child’s Nervous System.

[bib16] Fraser JA, Huang CLH (2004). A quantitative analysis of cell volume and resting potential determination and regulation in excitable cells. The Journal of Physiology.

[bib17] Gochberg DF, Kennan RP, Robson MD, Gore JC (1999). Quantitative imaging of magnetization transfer using multiple selective pulses. Magnetic Resonance in Medicine.

[bib18] Gochberg DF, Gore JC (2003). Quantitative imaging of magnetization transfer using an inversion recovery sequence. Magnetic Resonance in Medicine.

[bib19] Gudbjartsson H, Patz S (1995). The rician distribution of noisy MRI data. Magnetic Resonance in Medicine.

[bib20] Hamill OP, Marty A, Neher E, Sakmann B, Sigworth FJ (1981). Improved patch-clamp techniques for high-resolution current recording from cells and cell-free membrane patches. Pflugers Archiv.

[bib21] He B, Sohrabpour A, Brown E, Liu Z (2018). Electrophysiological source imaging: a noninvasive window to brain dynamics. Annual Review of Biomedical Engineering.

[bib22] Helfant RH (1986). Hypokalemia and arrhythmias. The American Journal of Medicine.

[bib23] Hennig J (1991). Echoes—how to generate, recognize, use or avoid them in MR‐imaging sequences: Part I: Fundamental and not so fundamental properties of spin echoes. Concepts in Magnetic Resonance.

[bib24] Hoffmann EK, Lambert IH, Pedersen SF (2009). Physiology of cell volume regulation in vertebrates. Physiological Reviews.

[bib25] Jefferys JGR (1995). Nonsynaptic modulation of neuronal activity in the brain: electric currents and extracellular ions. Physiological Reviews.

[bib26] Kamei H, Iramina K, Yoshikawa K, Ueno S (1999). Neuronal current distribution imaging using magnetic resonance. IEEE Transactions on Magnetics.

[bib27] Kane C, Shepherd RM, Squires PE, Johnson PRV, James RFL, Milla PJ, Aynsley-Green A, Lindley KJ, Dunne MJ (1996). Loss of functional KATP channels in pancreatic beta-cells causes persistent hyperinsulinemic hypoglycemia of infancy. Nature Medicine.

[bib28] Konn D, Leach S, Gowland P, Bowtell R (2004). Initial attempts at directly detecting alpha wave activity in the brain using MRI. Magnetic Resonance Imaging.

[bib29] Lang F, Busch GL, Ritter M, Völkl H, Waldegger S, Gulbins E, Häussinger D (1998). Functional significance of cell volume regulatory mechanisms. Physiological Reviews.

[bib30] Le Bihan D, Urayama SI, Aso T, Hanakawa T, Fukuyama H (2006). Direct and fast detection of neuronal activation in the human brain with diffusion MRI. PNAS.

[bib31] Le Bihan D (2012). Diffusion, confusion and functional MRI. NeuroImage.

[bib32] Lee SH, Han MJ, Lee J, Lee SK (2020). Experimental setup for bulk susceptibility effect‐minimized, multi‐orientation MRI of ex vivo tissue samples. Medical Physics.

[bib33] Lesage F, Lazdunski M (2000). Molecular and functional properties of two-pore-domain potassium channels. American Journal of Physiology. Renal Physiology.

[bib34] Li K, Zu Z, Xu J, Janve VA, Gore JC, Does MD, Gochberg DF (2010). Optimized inversion recovery sequences for quantitative T1 and magnetization transfer imaging. Magnetic Resonance in Medicine.

[bib35] Ling G, Gerard RW (1949). The normal membrane potential of frog sartorius fibers. Journal of Cellular and Comparative Physiology.

[bib36] Logothetis NK, Pauls J, Augath M, Trinath T, Oeltermann A (2001). Neurophysiological investigation of the basis of the fMRI signal. Nature.

[bib37] Luo Q, Lu H, Lu H, Senseman D, Worsley K, Yang Y, Gao JH (2009). Physiologically evoked neuronal current MRI in a bloodless turtle brain: detectable or not?. NeuroImage.

[bib38] Ma XY, Yu JM, Zhang SZ, Liu XY, Wu BH, Wei XL, Yan JQ, Sun HL, Yan HT, Zheng JQ (2011). External Ba2+ block of the two-pore domain potassium channel TREK-1 defines conformational transition in its selectivity filter. The Journal of Biological Chemistry.

[bib39] Mangia S, Chamberlain R, Martino F, Moeller S, Corum C, Kim T, Kalavagunta C, Michaeli S, Garwood M, Kim SG, Ugurbil K (2012). Functional MRI with SWIFT.

[bib40] Mansfield P (1977). Multi-planar image formation using NMR spin echoes. Journal of Physics C.

[bib41] Mathur-De Vré R (1980). The NMR studies of water in biological systems. Progress in Biophysics and Molecular Biology.

[bib42] McPhee KC, Wilman AH (2017). Transverse relaxation and flip angle mapping: Evaluation of simultaneous and independent methods using multiple spin echoes. Magnetic Resonance in Medicine.

[bib43] Miller KL, Bulte DP, Devlin H, Robson MD, Wise RG, Woolrich MW, Jezzard P, Behrens TEJ (2007). Evidence for a vascular contribution to diffusion FMRI at high *b* value. PNAS.

[bib44] Mostany R, Portera-Cailliau C (2008). A craniotomy surgery procedure for chronic brain imaging. Journal of Visualized Experiments.

[bib45] Neher E, Sakmann B (1976). Single-channel currents recorded from membrane of denervated frog muscle fibres. Nature.

[bib46] O’Donnell ME, Alvarez-Leefmans FJ, Delpire E (2009). In Physiology and Pathology of Chloride Transporters and Channels in the Nervous System.

[bib47] Ogawa S, Lee TM, Nayak AS, Glynn P (1990). Oxygenation‐sensitive contrast in magnetic resonance image of rodent brain at high magnetic fields. Magnetic Resonance in Medicine.

[bib48] Paley MNJ, Chow LS, Whitby EH, Cook GG (2009). Modelling of axonal fields in the optic nerve for direct MR detection studies. Image and Vision Computing.

[bib49] Parkes LM, de Lange FP, Fries P, Toni I, Norris DG (2007). Inability to directly detect magnetic field changes associated with neuronal activity. Magnetic Resonance in Medicine.

[bib50] Petridou N, Plenz D, Silva AC, Loew M, Bodurka J, Bandettini PA (2006). Direct magnetic resonance detection of neuronal electrical activity. PNAS.

[bib51] Phi Van VD, Sen S, Jasanoff A (2024). A different interpretation of the DIANA fMRI signal. Science Advances.

[bib52] Pottosin II, Bonales-Alatorre E, Valencia-Cruz G, Mendoza-Magaña ML, Dobrovinskaya OR (2008). TRESK-like potassium channels in leukemic T cells. Pflugers Archiv.

[bib53] Redmond L, Kashani AH, Ghosh A (2002). Calcium regulation of dendritic growth via CaM kinase IV and CREB-mediated transcription. Neuron.

[bib54] Roth BJ, Basser PJ (2009). Mechanical model of neural tissue displacement during Lorentz effect imaging. Magnetic Resonance in Medicine.

[bib55] Roth BJ (2023). Can MRI be used as a sensor to record neural activity?. Sensors.

[bib56] Stanisz GJ, Yoon RS, Joy MLG, Henkelman RM (2002). Why does MTR change with neuronal depolarization?. Magnetic Resonance in Medicine.

[bib57] Stroman PW, Lee AS, Pitchers KK, Andrew RD (2008). Magnetic resonance imaging of neuronal and glial swelling as an indicator of function in cerebral tissue slices. Magnetic Resonance in Medicine.

[bib58] Sundaram P, Wells WM, Mulkern RV, Bubrick EJ, Bromfield EB, Münch M, Orbach DB (2010). Fast human brain magnetic resonance responses associated with epileptiform spikes. Magnetic Resonance in Medicine.

[bib59] Sundaram P, Nummenmaa A, Wells W, Orbach D, Orringer D, Mulkern R, Okada Y (2016). Direct neural current imaging in an intact cerebellum with magnetic resonance imaging. NeuroImage.

[bib60] Tasaki I, Watanabe A, Sandlin R, Carnay L (1968). Changes in fluorescence, turbidity, and birefringence associated with nerve excitation. PNAS.

[bib61] Toi PT, Jang HJ, Min K, Kim SP, Lee SK, Lee J, Kwag J, Park JY (2022). In vivo direct imaging of neuronal activity at high temporospatial resolution. Science.

[bib62] Truong TK, Song AW (2006). Finding neuroelectric activity under magnetic-field oscillations (NAMO) with magnetic resonance imaging in vivo. PNAS.

[bib63] Truong TK, Roberts KC, Woldorff MG, Song AW (2019). Toward direct MRI of neuro‐electro‐magnetic oscillations in the human brain. Magnetic Resonance in Medicine.

[bib64] Tsien RY (1980). New calcium indicators and buffers with high selectivity against magnesium and protons: design, synthesis, and properties of prototype structures. Biochemistry.

[bib65] Vaughan PFT, Kaye DF, Ball SG, Reeve HL, Peers C (1995). The effect of barium on [3H]noradrenalin release from the human neuroblastoma SH-SY5Y. The European Journal of Neuroscience.

[bib66] Walz W (2000). Role of astrocytes in the clearance of excess extracellular potassium. Neurochemistry International.

[bib67] Wijesinghe RS, Roth BJ (2009). Detection of peripheral nerve and skeletal muscle action currents using magnetic resonance imaging. Annals of Biomedical Engineering.

[bib68] Xiong J, Fox PT, Gao JH (2003). Directly mapping magnetic field effects of neuronal activity by magnetic resonance imaging. Human Brain Mapping.

[bib69] Xu J, Li K, Zu Z, Li X, Gochberg DF, Gore JC (2014). Quantitative magnetization transfer imaging of rodent glioma using selective inversion recovery. NMR in Biomedicine.

[bib70] Zhou Y, Liu E, Müller H, Cui B (2021). Optical electrophysiology: toward the goal of label-free voltage imaging. Journal of the American Chemical Society.

